# Repetitive Transcranial Magnetic Stimulation for the Treatment of Resistant Depression: A Scoping Review

**DOI:** 10.3390/bs12060195

**Published:** 2022-06-17

**Authors:** Medard Kofi Adu, Reham Shalaby, Pierre Chue, Vincent I. O. Agyapong

**Affiliations:** 1 Department of Psychiatry, Faculty of Medicine and Dentistry, University of Alberta, 1E1 Walter Mackenzie Health Sciences Centre (WMC), 8440 112 St NW, Edmonton, AB T6G 2B7, Canada; rshalaby@ualberta.ca (R.S.); pchue@ualberta.ca (P.C.); vn602367@dal.ca (V.I.O.A.); 2 Department of Psychiatry, Dalhousie University, Halifax, NS B3H 4R2, Canada

**Keywords:** treatment-resistant depression, major depressive disorder, repetitive transcranial magnetic stimulation, mental health, treatment

## Abstract

Treatment-resistant depression (TRD) is associated with significant disability, and due to its high prevalence, it results in a substantive socio-economic burden at a global level. TRD is the inability to accomplish and/or achieve remission after an adequate trial of antidepressant treatments. Studies comparing repetitive transcranial magnetic stimulation (rTMS) with electroconvulsive therapy (ECT) and pharmacotherapy have revealed evidence of the therapeutic efficacy of rTMS in TRD. These findings suggest a crucial role for rTMS in the management of TRD. This article aims to conduct a comprehensive scoping review of the current literature concerning the use of rTMS and its therapeutic efficacy as a treatment modality for TRD. PubMed, PsycINFO, Medline, Embase, and Cinahl were used to identify important articles on rTMS for TRD. The search strategy was limited to English articles within the last five years of data publication. Articles were included if they reported on a completed randomized controlled trial (RCT) of rTMS intervention for TRD. The exclusion criteria involved studies with rTMS for the treatment of conditions other than TRD, and study and experimental protocols of rTMS on TRD. In total, 17 studies were eligible for inclusion in this review. The search strategy spanned studies published in the last five years, to the date of the data search (14 February 2022). The regional breakdown of the extracted studies was North American (n = 9), European (n = 5), Asian (n = 2) and Australian (n = 1). The applied frequencies of rTMS ranged from 5 Hz to 50 Hz, with stimulation intensities ranging from 80% MT to 120% MT. Overall, 16 out of the 17 studies suggested that rTMS treatment was effective, safe and tolerated in TRD. For patients with TRD, rTMS appears to provide significant benefits through the reduction of depressive symptoms, and while there is progressive evidence in support of the same, more research is needed in order to define standardized protocols of rTMS application in terms of localization, frequency, intensity, and pulse parameters.

## 1. Introduction

Major depressive disorder (MDD) is a mood disorder characterized by a depressed mood and/or a lack of interest or pleasure in previously rewarding or enjoyable activities, fatigue, disturbed sleep, the loss of appetite, and somatic and psychological symptoms [1,2]. MDD is a significant public health concern that affects approximately 300 million people globally, is a major leading cause of morbidity, and contributes immensely to the global burden of disease [3,4]. Effective treatment of MDD is available in the form of psychopharmacology, psychotherapy, electroconvulsive therapy (ECT), and other non-invasive brain stimulation methods [5], but affected patients frequently experience relapses and persistent life dysfunction [6], with associated suicidal ideation [7].

When a patient with MDD cannot attain remission or an adequate therapeutic response while being treated with one or more antidepressants, the patient is said to have developed treatment-resistant depression (TRD), and is diagnosed as such [8]. Because about 50 to 60% of MDD patients fail to attain a reasonable therapeutic response despite being treated with antidepressants, TRD is relatively common in clinical practice [9]. The most basic definition of TRD is the inability to accomplish and or achieve remission after an adequate trial of antidepressant treatment [9,10]. TRD is associated with delayed and high-cost inpatient times of treatment [10]. The suffering and disability associated with chronic, unremitting depressive illnesses are enormous, and TRD is considered to be responsible for the greatest healthcare burden associated with depressive disorders [11]. From the earliest conceptualization of TRD in 1974 [12,13,14], numerous studies have been conducted to determine the most effective treatment strategy for TRD [15,16].

As a result of the potential, high, direct and indirect medical costs, which further increase the severity of TRD, clinicians are in search of empirical evidence to guide in the choice of the most effective treatment for TRD [17]. A wide variety of treatment choices, including pharmacological and nonpharmacological interventions and somatic treatments, represent treatment options which are available for the management of TRD [18]. However, the decreasing therapeutic efficacy of antidepressant medications following at least two failed treatments, coupled with their potential side effects [19,20], has led to research into alternative treatment modalities, including repetitive transcranial magnetic stimulation (rTMS) [18].

As one of the current modes of treatment for MDD [21,22], the transcranial magnetic stimulation (TMS) technique was initially identified and developed by Barker et al. in 1985 [23]. Subsequently, other researchers modified the treatment technique to deliver TMS in repeated pulses in short intervals, which became known as rTMS [21]. rTMS has since been studied and evaluated by researchers for its potential therapeutic effect on many neurological and mental health conditions worldwide [24].

Studies comparing repetitive transcranial magnetic stimulation (rTMS) with electroconvulsive therapy (ECT) and pharmacotherapy have revealed evidence of the therapeutic efficacy of rTMS in TRD, and these findings suggest a key role of rTMS in the management of TRD [22]. An advantage of rTMS over other somatic treatments like ECT includes features such as not requiring anesthesia, and the fact that it can be delivered in an office setting, coupled with its having fewer treatment-associated side effects [25].

Approved by the US Food and Drug Administration (US FDA) for TRD [22], rTMS can be transmitted with either a low frequency (1 Hz) or a high frequency (10 Hz). While high-frequency rTMS is deemed to produce a stimulating effect on the cerebral cortex, low-frequency rTMS is believed to have an inhibitory effect [26]. There has been a steady increase in the stimulation dosages of rTMS application from early rTMS trials [27]. These increases include the stimulation intensity relative to the motor threshold and the number of pulses used in each treatment session. For instance, instead of the usual 10 to 20 trains of 10 Hz stimulation used for a high-frequency left-sided rTMS application [28,29], current trials apply up to about 75 trains for every treatment application daily [30,31]. This strategy has become the standard in many settings.

Studies suggest an imbalance in the efficient functioning of the frontal lobe in individuals diagnosed with depression [32]. Hence, researchers have treated patients with low-frequency rTMS to the right dorsolateral prefrontal cortex (DLPFC), or high-frequency stimulation to the left DLPFC [33,34]. It has been found that intermittent theta-burst stimulation (iTBS) delivered over 3 min is non-inferior to a standard 37.5 min treatment session at 10 Hz [35]. Furthermore, both low and high frequencies of rTMS application targeted to either the left or right DLPFC had the same therapeutic efficacy [36]. However, there were fewer side effects with the low-frequency right-sided application of rTMS [36].

The most effective treatment of TRD remains uncertain due to the limited validated pharmacological and psychotherapeutic approaches [37,38]. Given this limited evidence on the optimal treatment approach for TRD, rTMS has been evaluated as a treatment strategy [39]. Thus, increasing studies have been conducted that have focused on rTMS application in individuals diagnosed with TRD. The approval by the FDA for its use in the treatment of TRD reflects the evolving research on rTMS, for which the optimal technique of application continues to be investigated. rTMS is progressively becoming a common treatment modality, the parameters of which are still being defined. This review seeks to map an up-to-date synthesis of the currently available literature evidence supporting the therapeutic efficacy of rTMS in TRD while acknowledging that rTMS is a general approach rather than a single entity.

## 2. Methodology

In order to identify literature concerning rTMS for the treatment of TRD, five databases (PubMed, Embase, PsycINFO, CINAHL, and Medline) were electronically searched. The authors developed and executed a search strategy within the designated databases which included terms related to “treatment-resistant depression”, “repetitive transcranial magnetic stimulation”, “randomized control trials”, and “treatment”. The main aim of this review is to synthesize the evidence and assess the scope of current and updated literature on the use of rTMS in TRD. Furthermore, due the rapid advancement in this field with the use of newer techniques and parameters for rTMS applications, we opted to explore these recent updates in this review; therefore, the search strategy was limited to the last five years of data publication (from 2017 to February 2022). Language restrictions were applied, and only articles published in English were included. Two researchers independently conducted the title and abstract screening, and reviewed all of the full-text articles that met the inclusion criteria. Conflicts that arose out of the review process were discussed and resolved by the two reviewers. Table 1 displays the agreement of the two researchers in the full-text review.

We calculated Cohen’s Kappa Statistics, following the below equation, to report inter-rater reliability at the stage of the full-text review of the potential articles, where 0 = agreement equivalent to chance, (0.1–0.20) = slight agreement, (0.21–0.40) = fair agreement, (0.41–0.60) = moderate agreement, (0.61–0.80) = substantial agreement, (0.81–0.99) = near perfect agreement, and 1 = perfect agreement [40,41].

$$\mathrm{Kappa}=\frac{\mathrm{Observed}\;\mathrm{agreement}\;-\;\mathrm{chance}\;\mathrm{agreement}}{1\;-\;\mathrm{chance}\;\mathrm{agreement}}$$

### 2.1. Inclusion and Exclusion Criteria

Articles were included if they reported on a completed randomized controlled trial (RCT) of rTMS as a treatment intervention for TRD and were published within the last five years. The exclusion criteria involved studies with rTMS as a form of treatment for conditions other than TRD; studies and experimental protocols of rTMS on TRD were also excluded. Studies with rTMS as a combined therapy with pharmacotherapy or any other interventions were excluded, as were studies of rTMS treatment on treatment-resistant bipolar depression.

### 2.2. Data Extraction

A qualitative descriptive approach was used during the extraction to categorize the included studies based on the names of the authors, year of publication, study design, number of participants, targeted brain region, targeted symptoms, measurement tools, duration of treatment, coil/rTMS stimulations, outcome/significant improvements/effect size, assessment and follow-up, conclusion, and side effects of the interventionas displayed on Table 2.

## 3. Results

**Figure 1:**
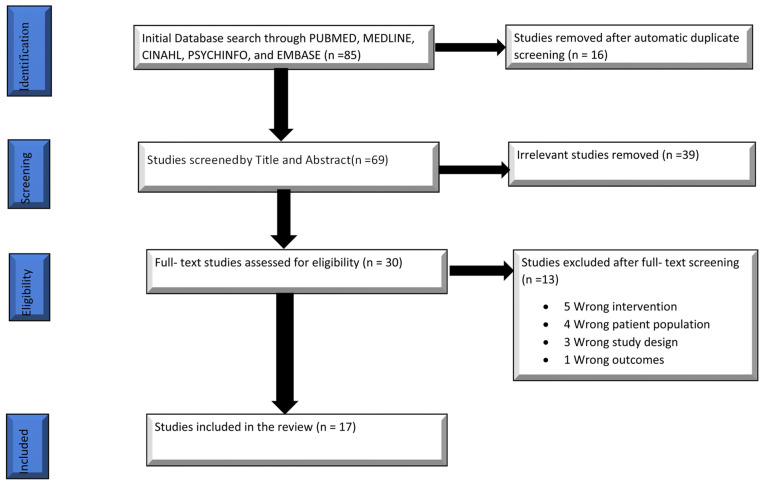
PRISMA flow chart describing the search results.

We identified 85 studies from the electronic databases through the search strategy and the use of the Covidence software. The software automatically screened and removed 16 duplicate studies from the searched items; 69 studies were screened against the eligibility criteria set based on the title and abstract only. The screening was performed independently by the two reviewers, and where conflicts in classification existed, the articles in question were discussed and a consensus was reached between the two reviewers. The title and abstract screening brought the total number of records left for full-text screening to 30 studies after 39 were deemed irrelevant, and were therefore excluded from the records. The remaining items were full texts screened by the two reviewers, and excluded 13 studies from the study. Studies were excluded primarily based on the wrong intervention, where the studies used CBT but were not specifically internet-based. In other studies, the target population had conditions other than TRD. There were studies with wrongful study designs, and some with wrongful outcomes. A total of 17 studies were legible and extracted for this scoping review. Figure 1 shows the PRISMA flow diagram displaying the search results and process.

Regarding the agreement of the researchers for full text review, Kappa analysis was conducted as shown in Table 1.

**Table 1:** Agreement of the two researchers in the full-text review.

		Researcher R.S.		
		Yes	No	Total
Researcher M.A.	Yes	15	4	19
	No	1	10	11
	Total	16	14	30

Observed agreement = 25/30 = 0.83

Chance agreement = (16/30) * (19/30) + (14/30) * (11/30) = 0.34 + 0.17 = 0.51

$$\mathrm{Kappa}=\frac{0.83\;-\;0.51}{1\;-\;0.51}=0.65$$

Kappa denotes a substantial agreement between the two researchers.

### 3.1. Overview of the Extracted Studies

Table 2 shows the extracted studies. Though the search strategy encompassed studies published in the last five years to the date of the data search (14 February 2022), we did not find any paper published in 2022 that met the inclusion criteria. Out of the 17 reviewed studies, we found n = 4, 23.5% each within 2019 and 2020, and n = 3, 17.6% from 2017, 2018, and 2021, respectively. Most of the studies were conducted in the USA (n = 7); Canada conducted two studies, and the UK, Greece, China, Netherlands, Australia, France, Croatia, and Japan all conducted one study each.

All 17 studies incorporated the RCT method, though in different formats and forms such as parallel, double-blind, open labels, and single-, two-, or four-arm forms. The sample size for the various trials ranged from n = 27 to n = 414. The participants in the various studies were all patients diagnosed with TRD or patients who had failed at least two adequate trials of different major classes of antidepressants. Out of the 17 papers, 15 were conducted in an adult population within the ≥18 age bracket. Two of the studies were conducted on older adults aged 60 and above. Only one study evaluated the effectiveness of rTMS in adolescents with a diagnosis of TRD.

### 3.2. Targeted Symptoms

All 17 studies evaluated the reduction in the severity of depression symptoms, the rate of responses or remissions, and the reduction on depression measuring scales. Several studies investigated other confounding factors that positively or negatively affect the results of rTMS. For instance, Carpenter et al. (2017) and Kavanaugh et al. (2018) targeted the effectiveness and safety of a two-coil rTMS device in their study subjects. Zhao et al. (2019), in their study, investigated the effects of rTMS on the serum levels of brain-derived neurotrophic factor, interleukin-1b, and tumour necrosis factor-alpha in elderly patients with refractory depression.

### 3.3. rTMS Protocol

In most studies (n = 7), the stimulation was conducted with a Magstim Super Rapid stimulator system [42,43,44,45,46,47,48]. Four studies also applied the MagProX-100 or R30 stimulator [35,49,50,51]. The NeuroStar XPLOR was utilized by two studies [52,53]. The MagVentureRX-100 [54], Magstim VR simulator [55], Medtronic MagPro 30 [27], and YRDCCY-1TMR [56] stimulators were applied in one study each. The Figure-8 coil was the most-commonly used (n = 7), followed by the B65-A/P coil (n = 4). The remaining studies used either the B70 fluid field-cooled coil or the 70-mm Double Air film coil.

### 3.4. Targeted Brain Region of rTMS

The brain site for rTMS application employed by the studies ranged from the left PFC (n = 5) [42,46,49,50,53] to the left DLPFC (n = 4) [35,48,52,56]. Four studies indicated DLPFC without specifying either the left or right [45,47,51,55], with one study evaluating the difference in effectiveness between the left and right DLPFC [27]. Two of the remaining studies [43,44] assessed the effectiveness of left DLPFC against dorsomedial PFC, and one study evaluated the differences in effectiveness between unilateral and bilateral left DLPFC [54].

### 3.5. Outcome Measures

A wide range of scales was used to measure positive symptoms and the reduction in symptom scales; for example, the Hospital Depression Rating Scale (HDRS) was the outcome measure in nine of the 17 studies, while the Hamilton Depression Scale (HAM-D) was used in six of the included studies. Other scales such as the Clinical Global Impression-Severity (CGI-S), Quick Inventory of Depressive Symptomatology (QIDS), Personal Health Questionnaire (PHQ-9), and Beck Depression Inventory (BDI-II) were also used to measure some of the primary or secondary outcomes in the included studies. Blumberger et al. (2018) defined their primary outcome as the reduction in the HDRS-17 score from the baseline to the end of treatment (either 20 or 30 treatments). If participants received most of the scheduled sessions and a 4-week, 5-week, or 6-week assessment was available, they were assessed for the primary endpoint. The safety outcomes included adverse event reporting, neurocognitive assessments, vital signs, and Columbia Suicide Rating Scale (C-SSRS) and Young Mania Rating Scale (YMRS) assessments for the various studies.

### 3.6. Outcome Results

Regarding the antidepressant efficacy of rTMS per the findings of this review, all 17 included studies evaluated its effectiveness and deemed it to be effective for the treatment of TRD, except for one study, in which the authors concluded that the standard 4-week rTMS treatment was not effective in chronic, severe TRD patients [46].

### 3.7. Efficacy of the Two-coil rTMS Device

An important observation made in this review is that it also included studies focusing on important confounding factors that either enhance or inhibit the efficacy of rTMS in patients with TRD. For instance, in their study, Kavanaugh et al. (2018) [43] sought to examine neurocognitive data from a randomized, double-blind, sham-controlled trial of an investigational two-coil rTMS device in TRD patients. The two-coil rTMS device is reported to stimulate deeper areas of the brain compared to the standard TMS devices, which primarily stimulate cortical brain areas, and may therefore have different adverse neurocognitive effects. The patients received 20 min daily rTMS with 10 Hz stimulation in the active and sham groups. The neurocognitive safety was evaluated at the baseline and within 72 h of the final treatment session. There were no observed negative neurocognitive effects of the two-coil rTMS device. The results revealed a significant effect of active rTMS on the quality of episodic memory; the baseline quality of episodic memory predicted depression treatment response and remission. The results were consistent with another RCT conducted by Carpenter et al. (2017), in which the researchers concluded that the delivery of rTMS with the two-coil device produced significant antidepressant effects after only 4 weeks of treatment and was well tolerated, with an effect size (Cohen’s d) f ITT d = 0.58; PP = 0.52 [44].

### 3.8. Tolerability and Side Effects

The overall effectiveness of any treatment intervention must acknowledge both its efficacy and regarding any safety and tolerability factors. In this regard, rTMS treatment appears to be reasonably well-tolerated, and the most common side effects were transient headaches, dizziness, and scalp discomfort at the stimulation site. However, Croarkin et al. (2021) [51] reported that one participant in both the sham group and active group developed suicidal ideation; the researchers classified this as not being related to the study device. In that same study, a patient was observed to have developed worsening depression during week four, and another had a suicide attempt during week six. Still, all of these adverse effects were classified as being unrelated to the study device. Yesavage, et al. (2018) [47] also reported cases of suicidal ideation in three active and four sham participants, though no suicides or seizures occurred during the study.

**Table 2:** Summary of studies using rTMS for the treatment of TRD.

Author (Year)	Country of Origin	Study Design	Age Range	Number of Participants	Targeted Brain Region	Targeted Symptom	Measurement	Duration of Treatment	Coil/ rTMS Parameters/Stimulation Method	Outcome/Significant Improvements/Effect Size	Assessment and Follow-Up	Conclusions	Side Effects
Rosen et al. (2021) [51]	USA	RCT	27–78 years	49	DLPFC	Change in depression symptoms	HAM-D 24 item	5–12 calendar days	MagPro R30 stimulator with a B65-A/P coil (10 Hz, 4 s on, 10 s off, 120% MT, 4000 pulses/session, 25 min per session) daily in blocks of 5 for a min. of 20 sessions (80,000 pulses), max. of 30 sessions (120,000 pulses)	Average stimulation location for responders vs. non-responders differed in the active but not in the sham condition (*p* = 0.02) Average responder location derived from the active condition showed significant negative functional connectivity with the subgenual cingulate (*p* < 0.001), while the non responder location did not (*p* = 0.17)	Baseline and acute phase	Clinical response to rTMS is related to accuracy in targeting the region within DLPFC that is negatively correlated with subgenual cingulate. Results support the validity of a neuro-functionally informed rTMS therapy target in veterans.	None reported
Theleritis et al. (2017) [42]	Greece	Parallel-group RCT	18–59 years	98	L-DLPFC	Change in depressive symptom severity	HDRS CGI-I	3 weeks	Magstim ultrarapid stimulator with a figure-8 magnetic coil. 40 trains of 20 Hz at 100% MT for 2 s and intertrain 1 min, yielding 1600 pulses per session	Twice-daily sessions might be more effective in both response and remission rates. Patients who had lower baseline HDRS (OR = 0.75, *p* = 0.014) and CGI-S scores (OR = 0.18, *p* = 0.001) were more likely to achieve remission	Baseline, and at the end of the first, second, third, and fifth week (follow up)	Twice per day, active HF-rTMS might be more effective than once per day, active HF-rTMS Practically none of the subjects in either sham group achieved remission	Discomfort at the site of stimulation Exacerbation of preexisting headache
Kavanaugh et al. (2018) [43]	USA	Double-blind, sham-controlled trial	18–70 years	84	L-DLPFC & dorso-medial PFC	Neurocognitive safety of the 2-coil device	HAM-D 24 CGI QLESQ-SF	4–6 weeks	2 Magstim Rapid2 stimulators. 70 mm figure-eight coil 10 Hz 120 MT of 4 s and 26 s rest Total of 3000 pulses per session	No observed negative neurocognitive effects of the 2-coil rTMS device. A significant effect of active rTMS was observed on the quality of episodic memory. Baseline quality of episodic memory predicted depression treatment response and remission.	Baseline, one month	2-coil rTMS device is a cognitively safe treatment for TRD that may possess episodic memory-enhancing capabilities.	Nil
Carpenter et al. (2017) [44]	USA	Randomized double-blind sham-controlled trial	18–70 years	92	L-DLPFC & dorso-medial PFC	Safety and efficacy of an investigational 2-coil rTMS device on depression symptoms	HAM-D 24 C-SSRS ATRQ	4–6 weeks	2 Magstim Rapid2 stimulators. single Magstim 70 mm figure eight coil 10 Hz 120 MT in trains of 4 s 26 s rest. 20 daily rTMS. A total 3000 pulses per session	n = 75 showed significantly greater improvement (mean HAMD-24 change) over time for the active (n = 38) versus sham (n = 37) group after 20 sessions (F = 7.174; *p* = 0.008) & also at the one-month follow-up (F = 6.748; *p* = 0.010) Effect size (Cohen’s d) for 4-week efficacy of rTMS with the two-coil device (ITT d = 0.58; PP = 0.52)	Baseline, Four weeks	Significant antidepressant effects after only 4-weeks of treatment and was well tolerated.	Headache Muscle twitch/spasms
Trevizol et al. (2019) [54]	USA	RCT	≥60 years	43	Unilateral & bilateral L- DLPFC	The primary outcome was the remission of depression.	HDRS SCID-II	3 weeks	Magventure RX-100 Stimulation with a cool B-65 figure-of-8 coil. 120% of RMT 10 Hz 15 sessions at five sessions/week over three weeks	Participants receiving bilateral rTMS experienced greater remission rates (40%) compared to unilateral (0%) or sham (0%) groups Response to rTMS in the HDRS similarly favoured the efficacy of bilateral rTMS	Baseline, week 3 week 6.	Sequential bilateral treatment may be an optimal form of rTMS when used for TRD in older adults	nil
DM Blumberger et al. (2018) [35]	Canada	Randomized non-inferiority trial	18–65 years	414	L-DLPFC	Change in the score of depression symptoms as read on HRSD-17	HRSD-17 QIDS-SR BSI-A DS-30	Five days a week for 4–6 weeks	MagPro X100 or R30 stimulator with B70 fluid-cooled coil. 10 Hz rTMS at 120% RMT 4 s on and 26 s off; 3000 pulses/session; total of 37.5 min. 120% RMT iTBS triplet 50 Hz bursts, repeated at 5 Hz; 2 s on and 8 s off; 600 pulses/ session; a total of 3 min 9 s	HRSD-17 scores improved from 23.5 (SD 4.4) to 13.4 (7.8) in the 10 Hz rTMS group and from 23.6 (4.3) to 13.4 (7.9) in the iTBS group (adjusted difference 0.103, lower 95% CI–1.16; *p* = 0.0011)	Baseline, after every five treatments and one week, Four weeks, and 12 weeks after treatment	iTBS is non-inferior to standard 10 Hz rTMS in reducing depressive symptoms.	Headache
Iwabuchi et al. (2019) [45]	Canada	RCT	18–70 years	27	DLPFC	rTMS Treatment response in TRD	HAM-D BDI	4 weeks	Magstim Super Rapid 2 Plus 1 stimulator 70 mm Double Air Film Coil. iTBS at ten bursts of 3 pulses 80%MT at 50 Hz applied at 5 Hz repeated at five runs of 600 pulses with 5 min rest. rTMS at 75 trains of 10 Hz 4 s per train rest 26 s intertrain intervals	rTMS treatment response rate was (55% for rTMS, 69% for iTBS). HAMD scores were significantly reduced at both one month (*p* < 0.001) and three months (*p* < 0.001) compared to baseline.	Baseline, Four weeks, 12 weeks	The study demonstrates that resting-state connectivity signatures can predict response to rTMS treatment in patients with resistant depression (irrespective of methodological variations in stimulus delivery).	Nil
BARBINI et al. (2021) [55]	UK	Randomized single-blinded study	-	80	DLPFC	Depressive symptoms in TRD	HDRS	3 weeks	rTMS applied MagstimVR stimulator with a figure-8 coil over the DLPFC.	rANOVA (F = 2.766, *p* = 0.043) & post-hoc in HDRS-17 showed significant better scores in favor of group B (rTMS plus BLT) every week (*p* < 0.025, T1: 22.075 vs. 17.200; T2: 16.100 vs. 12.775; T3: 12.225 vs. 8.900)	Baseline, week 1, week 2, week 3	The antidepressant effect of rTMS was enhanced and accelerated by its combination with BLT in treating resistant depression. Both treatment protocols were effective in reducing depressive symptomatology.	Nil
P.F.P. van Eijndhoven, et al. (2020) [46]	Netherlands	RCT	Adults	31	L-PFC	Depression symptoms in severe TRD patients	HDRS	4 weeks	Magstim Rapid 2 TMS with a focal, 8-figure shaped 70 mm coil. 110%RMT, 10 Hz 60 trains. 5 s with a resting period of 25 s between each train. 30 min with 3000 pulses/session, five days for four weeks, a total of 60,000 pulses	Interim analysis in the form of a mixed ANOVA indicated that there was a main effect of time (F (1,30) = 25.4; *p* < 0.01), but not for treatment (F(1,30) = 1.5; *p* = 0.23), and there was no interaction between time and treatment (F(1,30) = 0.45; *p =* 0.50)	Baseline, after 5, 10, 15, 20 sessions and one-week post-treatment	“Standard” 4-week rTMS treatment is not effective in chronic, severe TRD	Mild to moderate headache
Kito et al. (2019) [50]	Japan	Randomized open-label trial	25–75 years	30 (28 completed)	L-PFC	Remissions in depression symptoms	QIDS PHQ-9 YMRS	4–6 weeks	MagPro R30 magnetic stimulator and a Cool-B65 coil. rTMS at 120%MT, 10 HZ a total of 3000 pulses/d five days a week, for 4–6 weeks (Standardized rTMS) conventional rTMS 75 trains “4 s on and 26 s off” for 37.5 min with 3000 pulses	13/30 patients (43.3%) showed remission at week 6 There were no significant differences in the remission rate between the conventional 37.5-min and 18.75-min protocol groups (46.7% and 40.0%, respectively)	Baseline, week 2, week 4, and week 6.	Compared with conventional, rTMS with 18.75-min protocol might be equally effective and clinically beneficial in saving the treatment session length	Stimulation pain or discomfort
Filipčić et al. (2020) [47]	Croatia	Two-arm, unicentric, double-blind pilot randomized trial	18–68 years	28	DLPFC	Change in depression symptoms and rate of remissions	HDRS BDI-II	10–15 days	Magstim Rapid2 stimulator at 120% MT Each the session lasted for 20 min at 18 Hz: 2-s trains; 20-s intertrain intervals; 55 trains; a total of 1980 pulses per session or 3960 pulses per day	HDRS scores decreased by 13 (95% CI 11–17; 59%, 95% CI 45–73%) and 13 (95% CI 11–14; 62%, 95% CI 54–69%) points in the 10- and 15-day protocols, respectively	Baseline and daily adTMS	adTMS with H1-coil regimen twice daily for ten days or 15 days can be a safe and effective alternative for the treatment of TRD.	Nil
Benadhira, et al. (2017) [48]	France	Randomized sham-controlled study	22–79 years	58	L-DLPFC	Depression symptoms of TRD	HDRS	1 month (phase 1) 11 months (phase 11)	Magstim Super Rapid stimulator with figure-eight 70-mm coils 10 Hz at 110% MT 25 trains of 8 s interval of 30 s, for 5 days per week, for one month (20 sessions, M1) for a total of 2000 pulses per session.	Phase I, 35 patients were responders (60%) and 16 were partial responders (28%) 16 patients (28%) were in remission after one month of active rTMS HDRS scores, a significant difference was found between baseline and M1 (t (57) = 17.476; *p* < 0.001)	Baseline, weekly during the first month (M1) & monthly for the maintenance phase (M2 to M6)	rTMS could represent a novel strategy for preventing relapse in TRD patients who respond to rTMS treatment Weekly maintenance sessions could be useful, showing beneficial effects during the fourth month of treatment.	Nil
Roach et al. (2020) [52]	USA	Clinical trial	≥18 years	61	L-DLPFC	To test whether depressive symptoms changed significantly throughout treatment	PHQ-9	4–6 weeks	NeuroStar TMS 120% MT at 10 Hz 4 s followed by 10- to 26-s rest for a total of 3000 pulses/session. Five days a week for 4 to 6 weeks, for a total of 90,000 pulses	Average (SD) pretreatment and posttreatment PHQ-9 scores were 15.8 (6.2) and 12.6 (7.6), respectively. Statistically significant reduction in post–PHQ-9 was demonstrated (*p* < 0.001) with 69% of patients lowering their ratings & 31% demonstrating reliable change (improvement >5.64) Effect size (Cohen d = 0.46 on the paired t-test of pre–/post–PHQ-9)	Baseline, week 4, week 6	rTMS for TRD is an adequate treatment or augmentation option for ADSMs with MDD	Nil
Yesavage, et al. (2018) [49]	USA	A double-blind, sham-controlled randomized clinical	18–80 years	164	L-PFC	Remission of depression symptoms And the severity of depression symptoms	HRSD BDI	3 weeks	MagPro R30 device with Cool-B65-A/P coil. 10 Hz, 120%MT 5 sessions over 5 to 12 days A total of 4000 pulses/ session.	Overall remission rate was 39%, with no significant difference between the active and sham groups No significant effect of treatment (odds ratio, 1.16; 95% CI, 0.59–2.26; *p* = 0.67)	Baseline, end of treatment & 24-week follow up.	This study supports the clinical observation that a combination of interventions, including rTMS, effectively achieves symptom remission in 39.0% of veterans with MDD who were previously treatment-resistant.	Headache Naso- pharyngitis Suicidal ideation
Croarkin, et al. (2021) [53]	USA	Double-blind, randomized, sham-controlled trial	12–21 years	103 Sham (n = 55) Active (n = 48)	L-PFC	Change in the HAM-D 24 score	HAM-D, MADRS, CDRS-R, QIDS-A17-SR, CGI-S	6 weeks	NeuroStar XPLOR TMS 120%MT 10 pulses per sec (10 Hz) for 4 s, and with an interval of 26 s Each treatment session was 37.5 min (75 trains) for 3000 pulses per session.	Improvement in HAM-D-24 scores was similar between the active (−11.1 [2.03]) & sham groups (−10.6 [2.00]; *p* = 0.8; difference [95% CI], −0.5 [−4.2 to 3.3]) Response rates were 41.7% in the active group and 36.4% in the sham group (*p* = 0.6) Remission rates were 29.2% in the active group and 29.0% in the sham group (*p* = 0.95)	Baseline Week 4 and Weeks 6	Left prefrontal 10-Hz TMS monotherapy in adolescents with TRD is feasible, tolerable, and safe A statistically significant difference between 6 weeks of sham and active TMS was not observed.	Suicidal ideation, worsening depression during week 4, suicide attempt during week 6
Fitzgerald et al. (2020) [27]	Australia	Four arm RCT	Adults	300	L-DLPFC & R DLPFC	Response and remission rates of depression symptoms	HRSD-17	4 weeks	Medtronic Magpro30 magnetic stimulators with fluid-filled 70 mm figure-of-8 coils rTMS at 120% RMT 10 Hz for groups (1 and 2), 1 Hz for groups (3 and 4). (left standard = 50 trains, left high = 125 trains, right standard = 20 min, right high = 60 min, all per day in a single session).	The rate of response exceeded 45% in all groups No significant difference between groups on initial analysis of the primary or secondary outcome measures (response rates: standard left = 52.5%, high left = 47.3%, standard right = 49.1%, high right = 48.4%) Greater remission rate with high compared to moderate dose left- sided treatment when controlling for illness duration	Baseline and after 1, 2, 3, and 4 weeks	No consistent association between the antidepressant effect of rTMS & the number of TMS pulses provided across the ranges investigated in this study. Increasing TMS pulse number in individual sessions seems unlikely to be a method to substantially improve clinical outcomes.	Nil
Zhao et al. (2019) [56]	China	RCT	≥60 years	58	L-DLPFC	Serum levels of brain-derived neurotrophic factor (BDNF), interleukin (IL)-1b, and tumour necrosis factor (TNF)-a in elderly patients with refractory depression.	HAM-D 24	1 month	YRDCCY-I TMR apparatus 10 Hz at 80% MT	BDNF levels gradually increased with treatment duration in the rTMS group and were significantly higher compared with the control group In contrast, IL-1b and TNF-a levels gradually decreased and were significantly lower than in the control group None of the serum factors was affected by rTMS in healthy individuals	Baseline, at 48 h and 1, 2, 3, and 4 weeks after the first TMS treatment	rTMS increased serum BDNF levels and decreased serum IL-1b and TNF-a levels in patients with depression but had no effect on any of these factors in healthy individuals Results suggest that rTMS may increase BDNF and decrease IL-1b and TNF-a serum levels in elderly patients with refractory depression.	Nil

MT = Motor Threshold, SMA = Supplementary Motor Area; HAM-D 24 = Hamilton Rating Scale for Depression—24 item; BDI–II = Beck Depression Inventory; DLPFC = Dorsal Lateral Prefrontal Cortex; OFC = Orbitofrontal Cortex; RMT = Resting Motor Threshold; CGI-I = Clinical Global Impression; HAM-A = Hamilton Anxiety Rating Scale; HRSD = Hamilton Rating Scale for Depression; YMRS = Young Mania Rating Scale; GAF = Global Assessment of Functioning; MCCB = MATRICS Consensus Cognitive Battery; QIDS = Quick Inventory of Depressive Symptomatology; BNCE = Brief Neurobehavioral Cognitive Examination Questionnaire; SCID = Structured Clinical Interview for the DSM-IV; IPF = Inventory of Psychosocial Functioning; BRMAS = Bech–Rafaelsen mania scale; CRSD = Circadian Rhythm Sleep Disorder; SCL-90-R = Symptom Checklist-90-Revised; mPFC = Medial Prefrontal Cortex.

### 3.9. Frequency, Intensity of Stimulation, and Duration of Treatment

The frequency of rTMS ranged from as low as 5 Hz to as high as 50 Hz. The majority of the studies (13 out of 17) applied the 10 Hz frequency, and two studies applied the 50 Hz frequency. The intensity of stimulation reviewed in the included studies also ranged from the 80% to the 120% motor threshold, but most of the studies (11) applied the 120% motor threshold in their investigations. The duration of active rTMS treatments in the included studies ranged from 3 weeks to 6 weeks, while the only maintenance treatment reviewed lasted for about 11 months. Concerning the number of magnetic pulses given per treatment session, there was a range varying from 600 pulses to 4000 pulses.

### 3.10. Variations in the Brain Target

Accuracy in targeting functional brain networks is deemed essential for the treatment efficacy of rTMS in TRD. One study tested whether variations in targeting precision contributed to the failure to find an advantage of active over sham treatments [51]. In this study, the researchers used data from a failed clinical trial of rTMS in veterans to test whether treatment response was associated with the rTMS coil location in the active but not sham stimulation, and compared fMRI functional connectivity between those stimulation locations. The results indicated that the response to rTMS was related to accuracy in targeting the region within DLPFC that is negatively correlated with subgenual cingulate.

### 3.11. Comparing the Efficacy and Tolerability of the Different Forms of rTMS

In order to establish the true efficacy of rTMS in depression-related conditions, current studies are beginning to focus attention on the different forms of rTMS, and are comparing their effectiveness and tolerability to the standard rTMS. For instance, Blumberger et al. (2018) [35] aimed to evaluate the clinical effectiveness, safety, and tolerability of iTBS compared with the standard 10 Hz rTMS in adult treatment-resistant depression patients. The participants were randomized to receive iTBS or 10 Hz rTMS. Both groups were assessed at 4–6 weeks for the primary outcome. The HRSD-17 scores for the 10 HZ rTMS improved from a baseline of 23.5 (SD 4.4) to 13.4 (7.8), and from 23.6 (4.3) to 13.4 (7.9) in the iTBS group. The adjusted difference was 0.103 (lower 95% CI–1.16; *p* = 0.0011). The conclusion was that iTBS is non-inferior to standard 10 Hz rTMS in reducing depressive symptoms in TRD patients, with the advantage that the utilization of iTBS can increase the number of patients treated in a day without affecting the clinical efficacy of the treatment.

### 3.12. Maintenance rTMS Treatment

Regarding the efficacy of maintenance rTMS after an acute response in depression, Benadhira et al. (2017) [48] evaluated the role of maintenance rTMS in TRD patients who responded to one month of active rTMS in an open-labelled study (phase I). They assessed the benefits of a randomized protocol of maintenance rTMS for up to eleven months (phase II). Clinical assessment was at the baseline, weekly during the first month, and then monthly for the maintenance phase. The results indicated that the antidepressant effect of maintenance rTMS sessions appeared three months after the treatment (Month 4). Maintenance rTMS was well tolerated, and no side effects were reported. The study suggests that rTMS could represent a novel strategy for reducing relapse in TRD patients who respond to rTMS treatment. This result contrasts a trial in which patients were randomized to once-a-month rTMS maintenance treatment and an observation-only group, the results of which failed to predict any statistically significant difference between the two groups at the end of a 1-year study period [57].

### 3.13. Relationship between the Pulse Number and the Response to rTMS in TRD

There has been a steady increase in the stimulation dosage of rTMS application from the early stages of rTMS trials to date. These increases include the stimulation intensity relative to the motor threshold and the number of pulses used in each treatment session. However, very few studies have sought to evaluate the differences in pulse numbers and the response to rTMS in patients. Fitzgerald et al. (2020) [27] investigated whether the response to rTMS is greater when it is applied at a higher pulse than a lower pulse. The participants were grouped into four treatment groups:

1. Standard-dose HFL-rTMS: 50 trains of 10 Hz rTMS; 4.5 s trains at 120% RMT with a 20.5 s inter-train interval (2250 pulses/session).
2. High-dose HFL-rTMS:125 trains of 10 Hz rTMS; 4.5 s trains at 120% RMT; a 15.5 s inter-train interval (5625 pulses/session).
3. Standard-dose LFR-rTMS: one continuous train of 1 Hz rTMS; 20 min at 120% RMT (1200 pulses).
4. High-dose LFR-rTMS: two trains of 1 Hz rTMS; 30 min at 120% RMT (3600 pulses/session).

The treatment was applied for four weeks, five days/week, for 20 treatment sessions. In terms of results, there was no consistent association between the antidepressant effect of rTMS and the number of TMS pulses across the ranges. Thus, increasing the TMS pulse in individual sessions did not seem to be a potential method to substantially improve clinical outcomes.

### 3.14. Effect of rTMS on the Serum BDNF, IL-1b, and TNF-a Levels in TRD

Inflammatory factors such as interleukin (IL)-1 [58], tumor necrosis factor (TNF)-a [59], nuclear factor-kappaB (NF-jB) [60], and brain-derived neurotrophic factor (BDNF) have been implicated in the causative mechanism of depression [61]. However, there are limited studies on the specific effects of rTMS on these inflammatory factors in patients with TRD. In the study by Zhao et al. (2019) [56], elderly depressed patients were randomized into two groups of 29, with one group receiving rTMS and the other as a control group, while another group of 30 healthy volunteers were given rTMS. The serum levels of BDNF, IL-1b, and TNF-a were measured before the study and at 48 h, and 1, 2, 3, and 4 weeks after the first TMS treatment. rTMS increased serum BDNF levels and decreased serum IL-1b and TNF-alpha levels in patients with depression, but it had no effect on any of these factors in healthy individuals.

## 4. Discussions

The studies included in this review were RCTs published within the last five years, between 2017 and 2022 (though none of the eligible studies were extracted from 2022). Overall, these studies are characterized by their varying sample sizes, ranging from small to large, and are heterogeneous in terms of their demographic and clinical variables, and in terms of their choices of brain targets of rTMS stimulation, treatment duration, and stimulus intensity. The 17 studies reviewed here suggest that rTMS appears to have a robust therapeutic effect in the treatment of TRD. The regional breakdown of the extracted studies revealed that most studies (n = 9) were conducted in North America. Depression is a global burden and a debilitating condition that exacts a serious personal, social, and economic toll [62]; it is associated with extreme consequences such as increased mortality, disability, and secondary morbidity [63]. The World Health Organization has recently reported that depression ranks among the leading causes of disability worldwide [64].

All but one study [46] reported consistent improvements in depressive symptoms through higher or accelerated doses and patient-centred stimulation protocols across the major outcome domains. These positive outcomes were enhanced by accurate and advanced neuro-navigational technologies, the degree of precision in the techniques of the detection of the DLPFC, and the application of modern coil geometries. Because rTMS treatment is rapidly gaining popularity as a treatment modality for TRD, there should be a focus of attention on global accessibility, reliability, and efficacy through standardized protocols and evidence-based guidelines.

Though the primary objective of all 17 studies was the reduction and remission of depressive symptoms in TRD patients, some of the studies evaluated other confounding factors that affect the efficacy of rTMS intervention in the management of TRD. Two out of the 17 studies evaluated a two-coil rTMS device [43,44]. Though the antidepressant mechanism of multi-coil stimulation and whether it differs from that of standard single-coil stimulation are still being investigated, studies have reported that the depth and direction of the electromagnetic field capable of penetrating the scalp and tissues of the brain for the activation of neurons during the process of rTMS application are dependent on the shape and size of the coil through which the current is passed [30,31]. Until recently, most rTMS depression interventions were performed using figure-of-eight or butterfly-shaped coils deemed to emit relatively superficial cortical stimulations. However, the pathophysiology of depression is assumed to involve a variety of deeper frontal brain regions [65,66]. Therefore, the two-coil rTMS device was specifically designed to target brain pathways for possible deeper cortical stimulations, and may represent a novel technique for neurostimulation for patients with TRD.

There were limited data on maintenance rTMS treatment for TRD. Only one out of the 17 reviewed papers evaluated the efficacy of maintenance rTMS after an acute response in the treatment of TRD. Their results indicated that the antidepressant effect of maintenance rTMS sessions appeared three months after the treatment. Maintenance rTMS was well tolerated, and no side effects were reported [48]. This result contrasts with an earlier study that investigated 12-month outcomes comparing two maintenance TMS approaches: a scheduled, single TMS session delivered monthly versus an observation-only group, which found that there were no significant group differences in any outcome measure [57]. This suggests that although rTMS could represent a novel strategy for reducing relapse in TRD patients who respond to rTMS treatment, there is little information on its maintenance use. As explained in the literature, maintenance treatment is not the mere reintroduction of rTMS in situations of a relapse; rather, it is an intentional, timely, scheduled regimen of rTMS treatment for a fixed period after acute rTMS treatment [21]. Much more research needs to be conducted, and the true effect of maintenance rTMS treatment in TRD must be ascertained.

Regarding brain targets, the DLPFC was the most frequent (n = 9) rTMS site targeted with the primary preference for the left DLPFC; none of the studies applied rTMS to the right DLPFC. Only one study compared the relationship between the pulse number and the response to rTMS in depression between the left and right DLPFC [27]. The left PFC was also utilized in six studies, which reported improvement in depressive symptoms. The left DLPFC represents an essential brain region for neurocognitive performance connecting to the frontosubcortical brain regions [67]. The dysfunctions of this brain region are believed to be involved in the pathogenesis of symptoms of depression and cognitive impairment [68,69]. The stimulation of the DLPFC is significantly associated with the enhancement of the neurocognitive domains, and rTMS appears to reduce depressive symptoms, with a subsequent improvement in the neurocognitive functions of TRD patients [30,70,71].

According to our findings, all 17 of the reviewed studies applied rTMS with a high frequency—ranging from 18 Hz to 50 Hz—in their subjects. Studies have it that the effectiveness of rTMS treatment regarding the modulation of neural activities greatly depends on the frequency applied and other stimulation parameters [72]. High-frequency rTMS over the DLPFC has been used in the most recent trials—a choice guided by the positive outcome results for this approach [73]. This possibly explains the positive outcomes brought about by our reviewed studies, as the rTMS targets were mostly the left DLPFC with high frequencies. Again, our results revealed a trend in which all of the included papers applied rTMS with high stimulus intensity ranging from 80 MT to 120 MT. Though not all RCTs that apply higher stimulating intensities end up with larger effect sizes, stimulus intensity is deemed to be an essential component in the induction of lasting changes in cortical excitability, which is believed to be responsible for the antidepressant effect of rTMS [73]. This report is consistent with our findings, as all of the studies applied high stimulating intensities and still had the desired treatment effects.

Overall, rTMS treatment in the management of TRD seems safe and tolerable. All 17 studies reported on the treatment side effects and tolerability of rTMS. The most common side effects across all of the studies were scalp pain, transient headaches, dizziness, and discomfort at the stimulation site, but these side effects did not lead to the discontinuation of the treatment. However, two studies reported cases of suicidal ideation and a worsening in depressive symptoms, though no suicides or seizures occurred during the treatments [49,53]. Consistent with data from earlier studies [74,75,76,77], our results add to the evidence that supports the safe and tolerable nature of rTMS in TRD.

### 4.1. Cost and Policy Implications for rTMS in TRD

The global burden of disease study 2010 ranked MDD as the second leading cause of disability globally, accounting for an estimated 2.5% of global disability-adjusted life-years and 8.2% of global years lived with disabilities [78]. Among the many treatment modalities for the management of TRD, rTMS is considered to be a clinically safe, productive, and patient-preferred treatment modality in resistant depression. However, the treatment benefits of rTMS need to be weighed against its treatment-related cost. A study evaluated the cost-effectiveness of rTMS vs. ECT for TRD from Singapore’s societal perspective. The results demonstrated that, compared to ECT, rTMS was associated with lower total cost (SGD 23,072 vs. SGD 34,922) and Quality-Adjusted Life Years (QALYs) (0.6862 vs. 0.7243) over one year. Thus, rTMS was considered to be highly cost-effective relative to ECT [79]. Their result was consistent with a prospective economic evaluation of ECT and rTMS in the United States. The model provided support for the economic benefit of rTMS versus ECT alone in non-psychotic depression. Their results revealed that the cost of the acute treatment of rTMS was $1422.00, versus $7758.40 for ECT [80].

The comparative cost-effectiveness can help to inform decisions on resource allocation and treatment utilization. Globally, healthcare resources are mostly scarce relative to needs or wants, and the essence of an economic evaluation is to inform the choices that decision-makers face in critical situations. However, there is a paucity of literature on the cost-utility analysis of TRD management. Therefore, investigating the resource implications and cost-effectiveness of rTMS offers crucial information that may help the choice of treatment for people with treatment-resistant depression. Future studies should focus on the cost–benefit analysis of rTMS in TRD.

### 4.2. Limitations

There are several limitations to this review. One main limitation relates to the small number of studies that were included for qualitative synthesis and analysis. However, our search strategy considered only studies published in English within the last five years (2017–2022). Secondly, although we carefully tried to identify all of the necessary studies for this review per our eligibility criteria, we still may have missed some relevant studies, particularly those published in other languages. Finally, the eligibility criteria only took into account RCTs, and furthermore, no meta-analysis was run on the reported data.

## 5. Conclusions

rTMS treatment is progressively gaining popularity in the treatment of depressive conditions, and there is evidence in support of the efficacy of rTMS in TRD. The treatment is considered effective, safe, and tolerable in the management of TRD. However, while progressive evidence supports its efficacy in an acute setting, there is limited literature to support long-term benefits and maintenance treatment in patients with TRD. Large-scale clinical trials are needed to compare the therapeutic efficacy and efficiency of the newer forms of rTMS with the consistency of the stimulating parameters across all of the treatment arms. Finally, in order to be able to establish a standardization of rTMS application, more studies are required to address frequency, intensity, pulse numbers, and localization.
